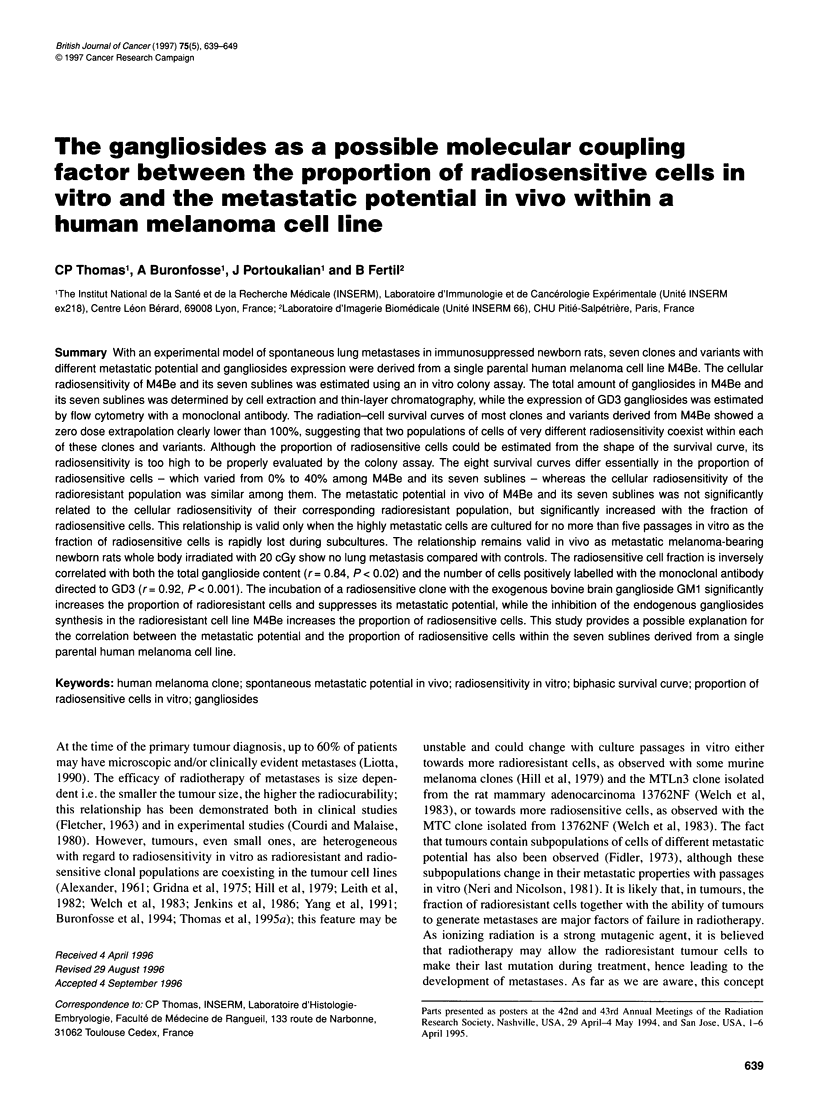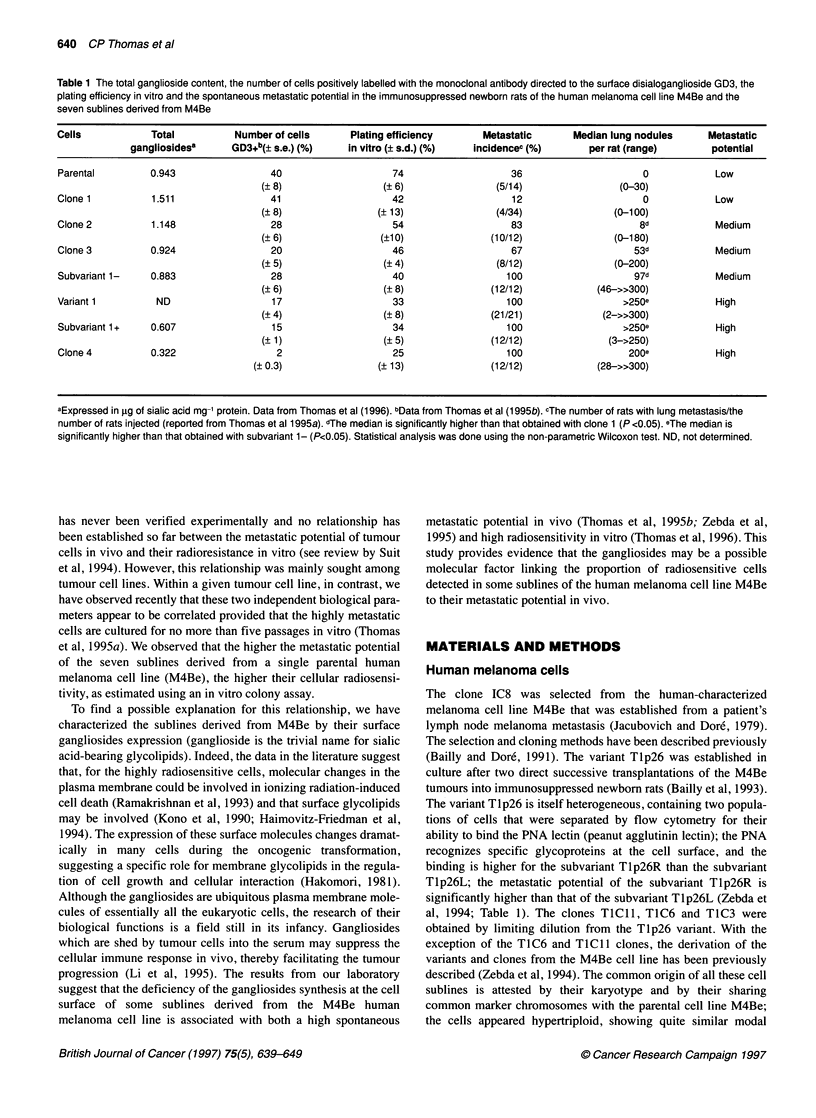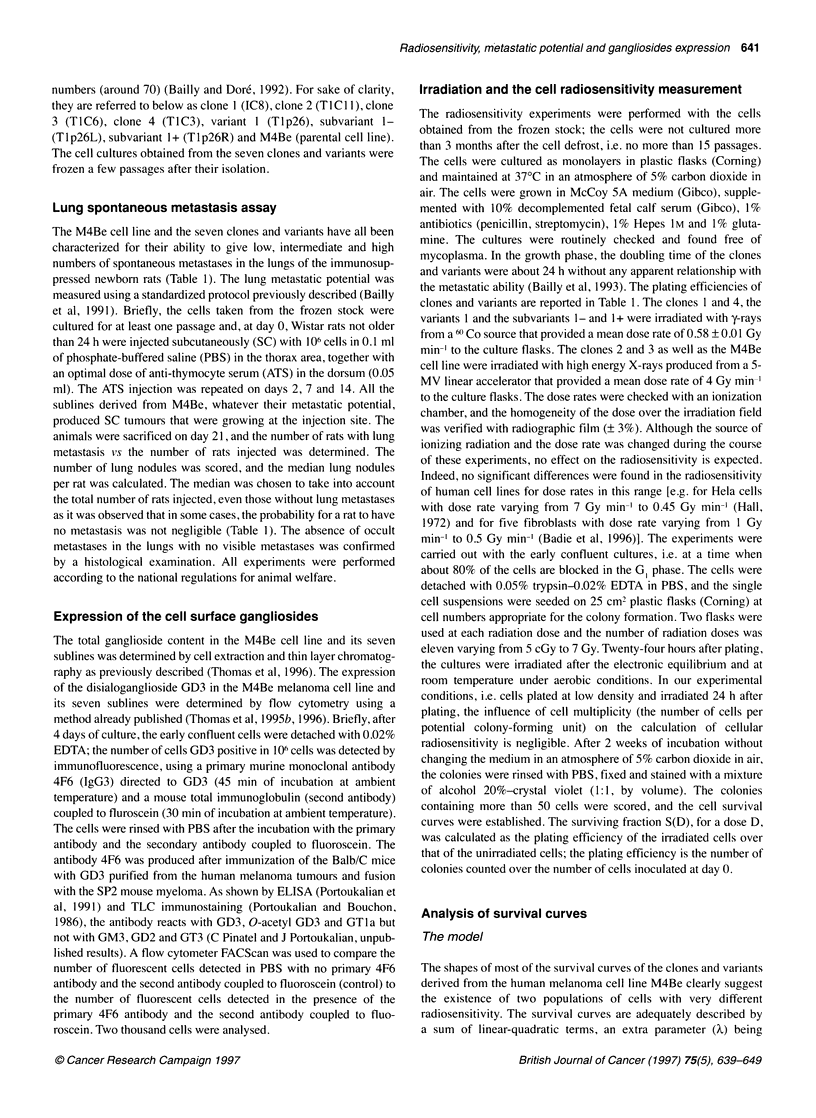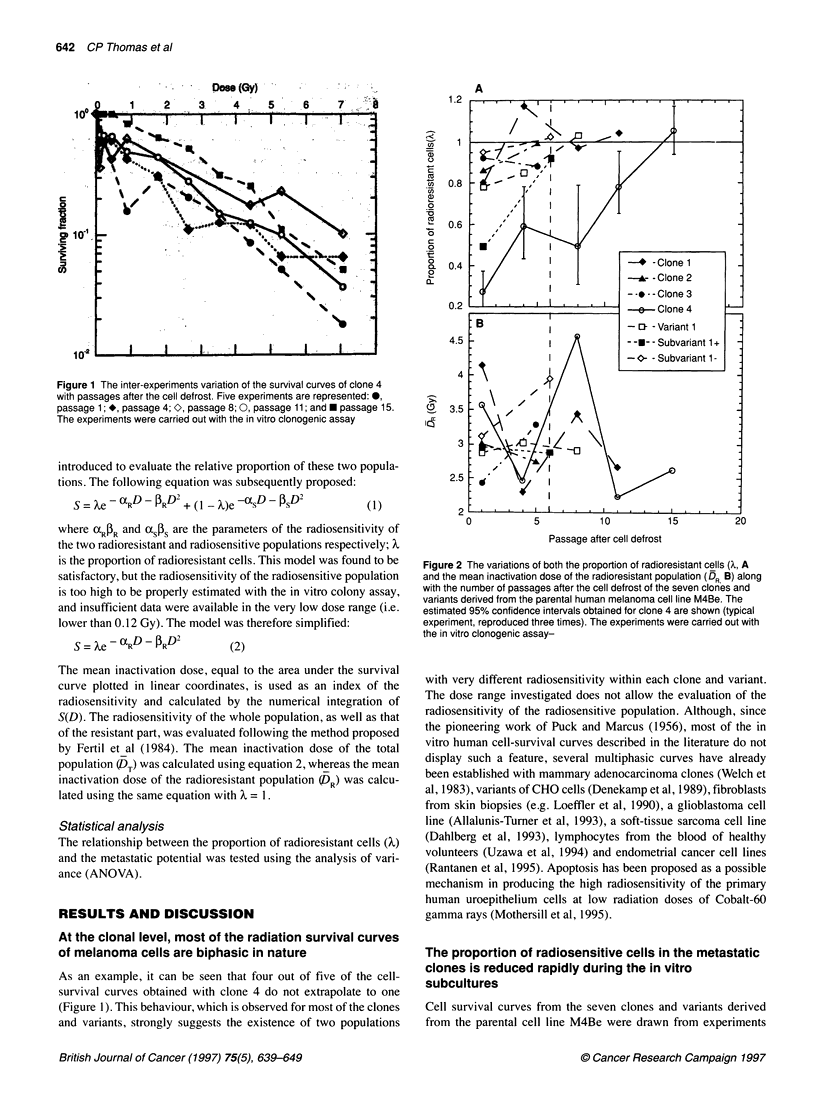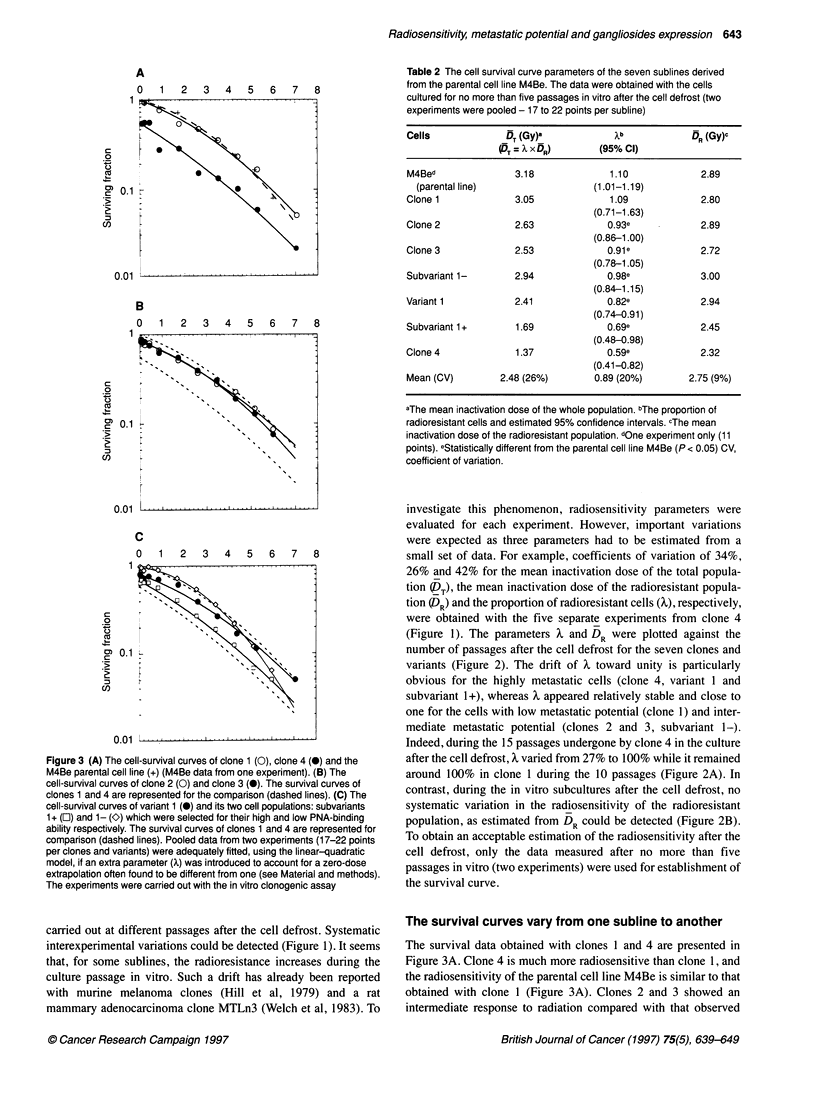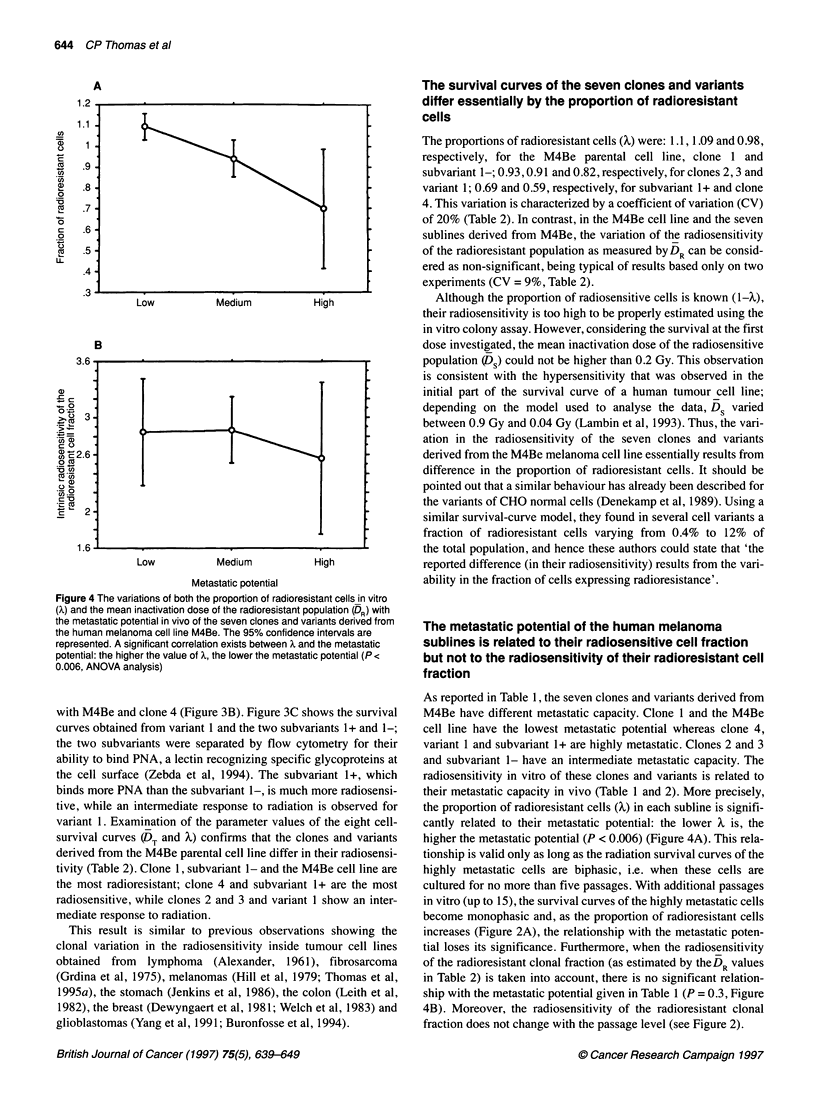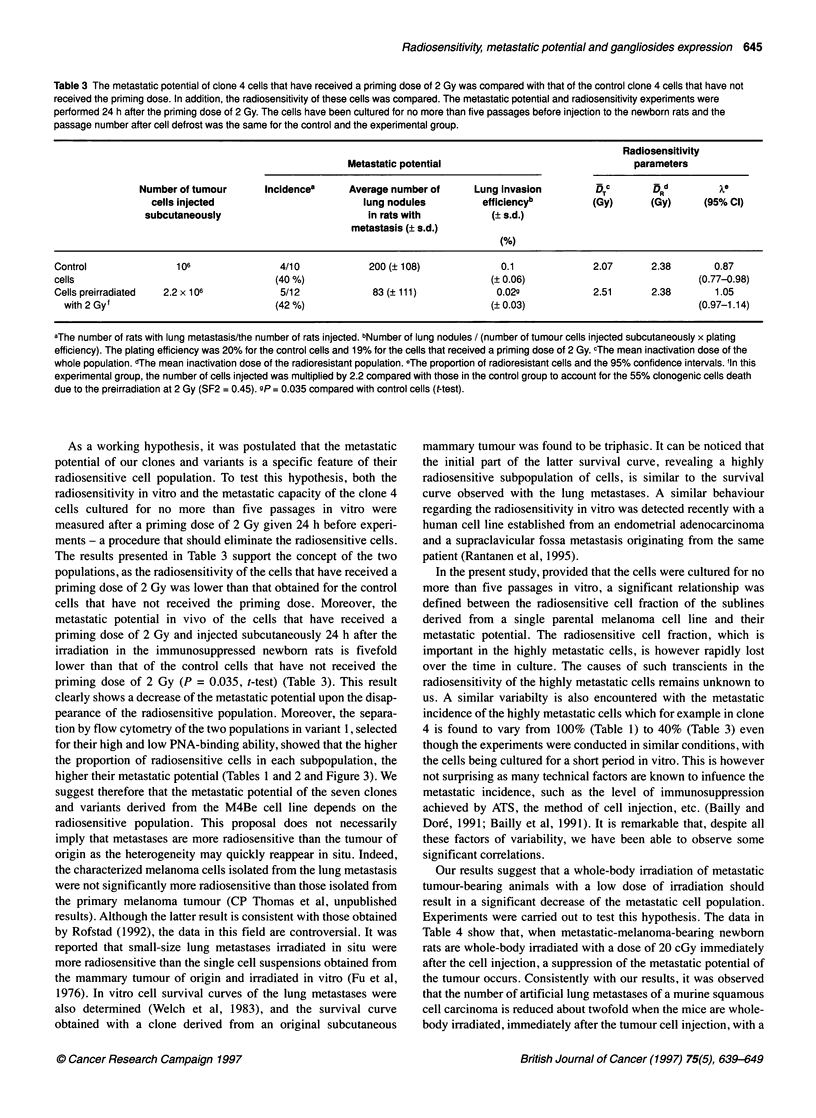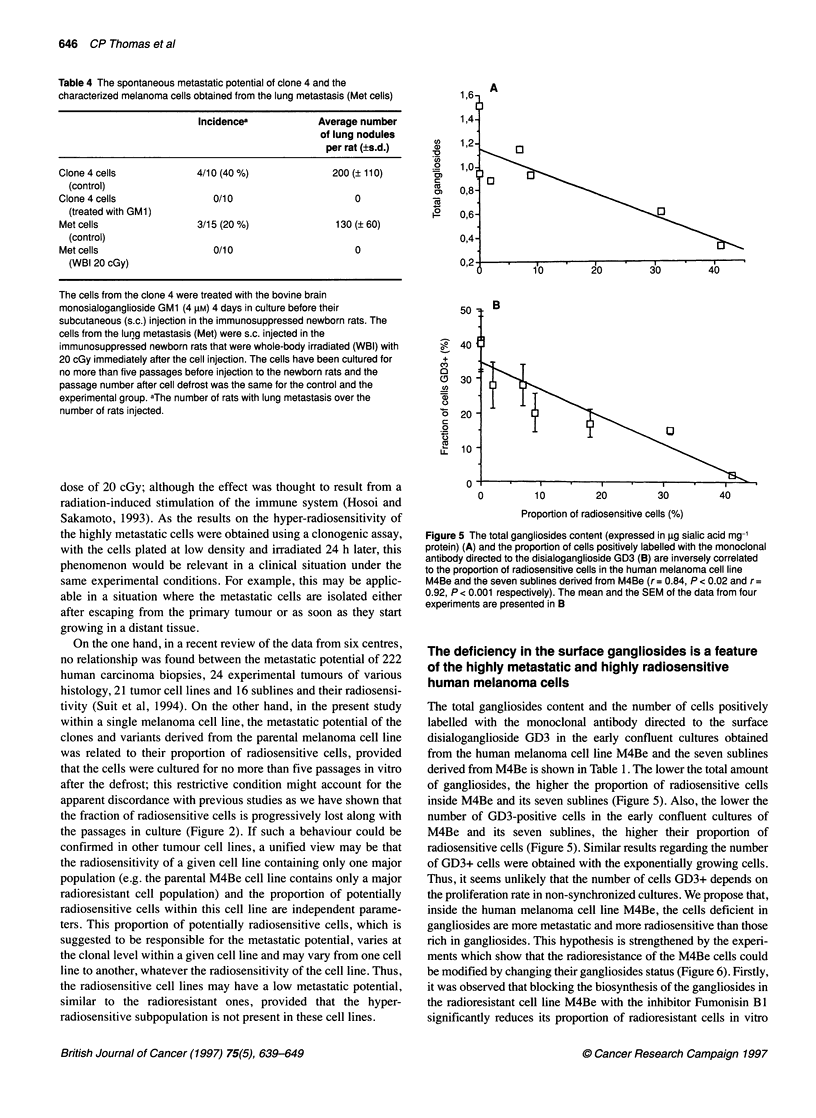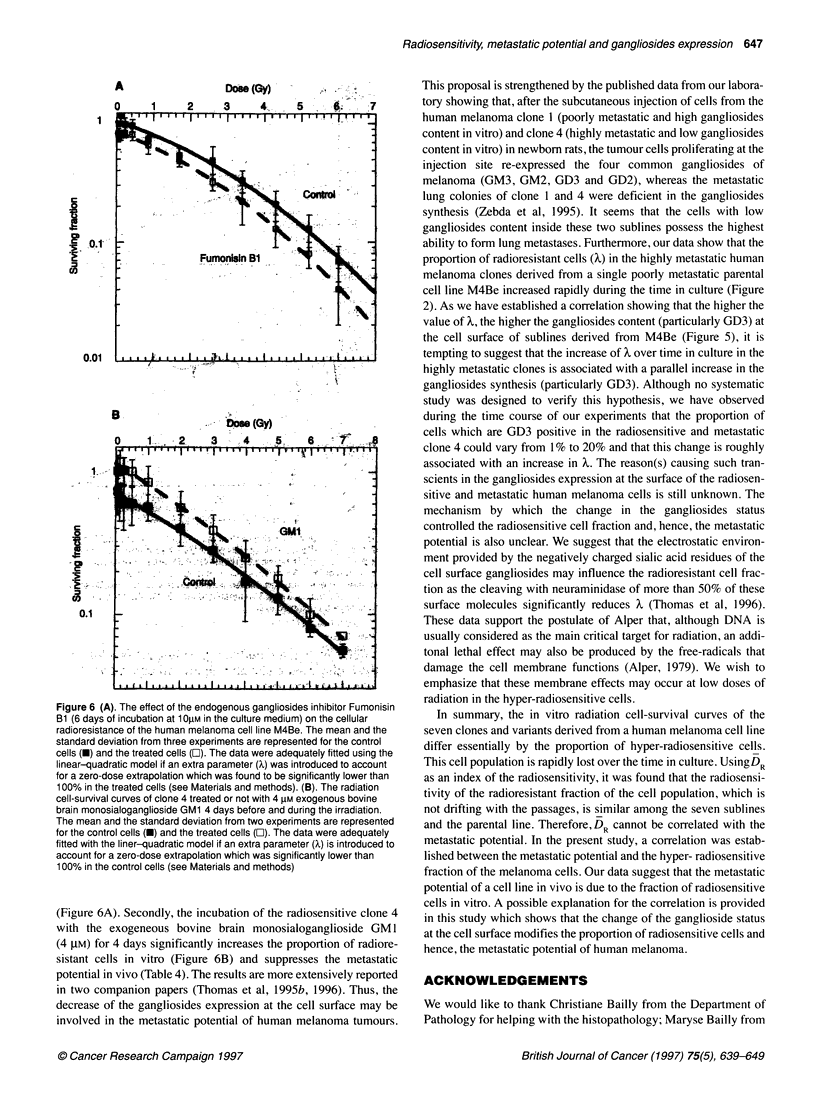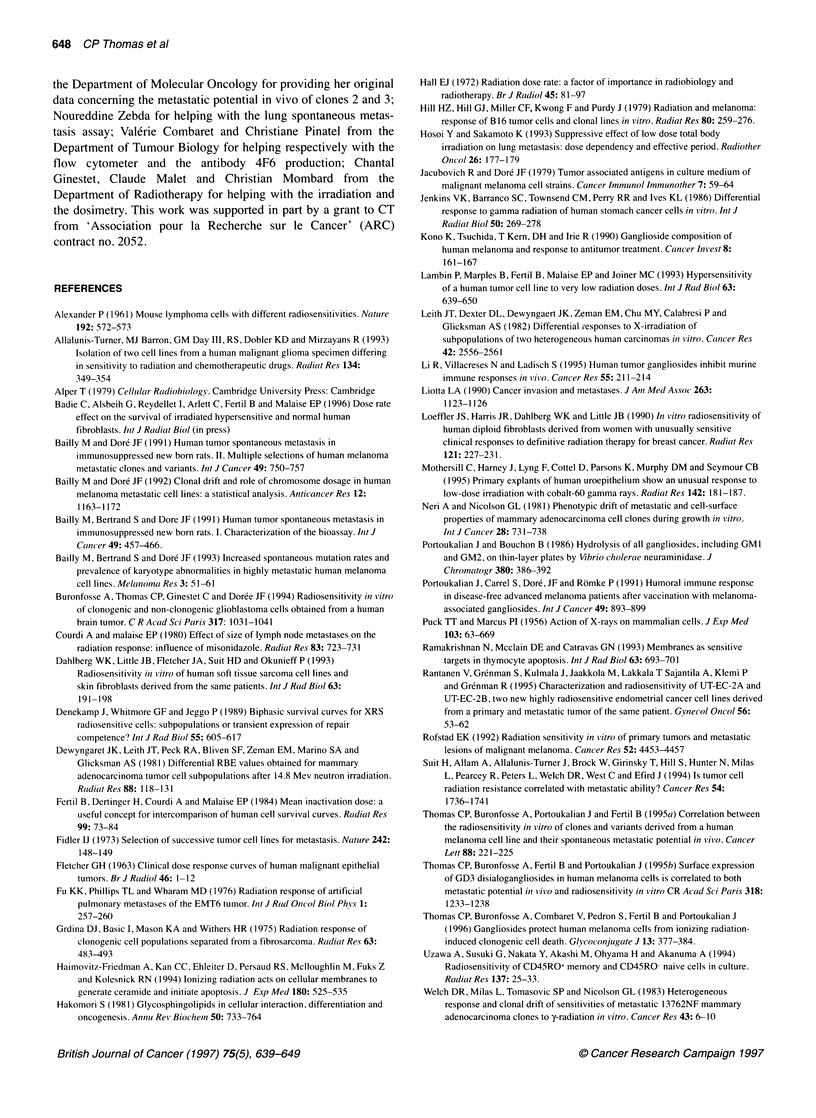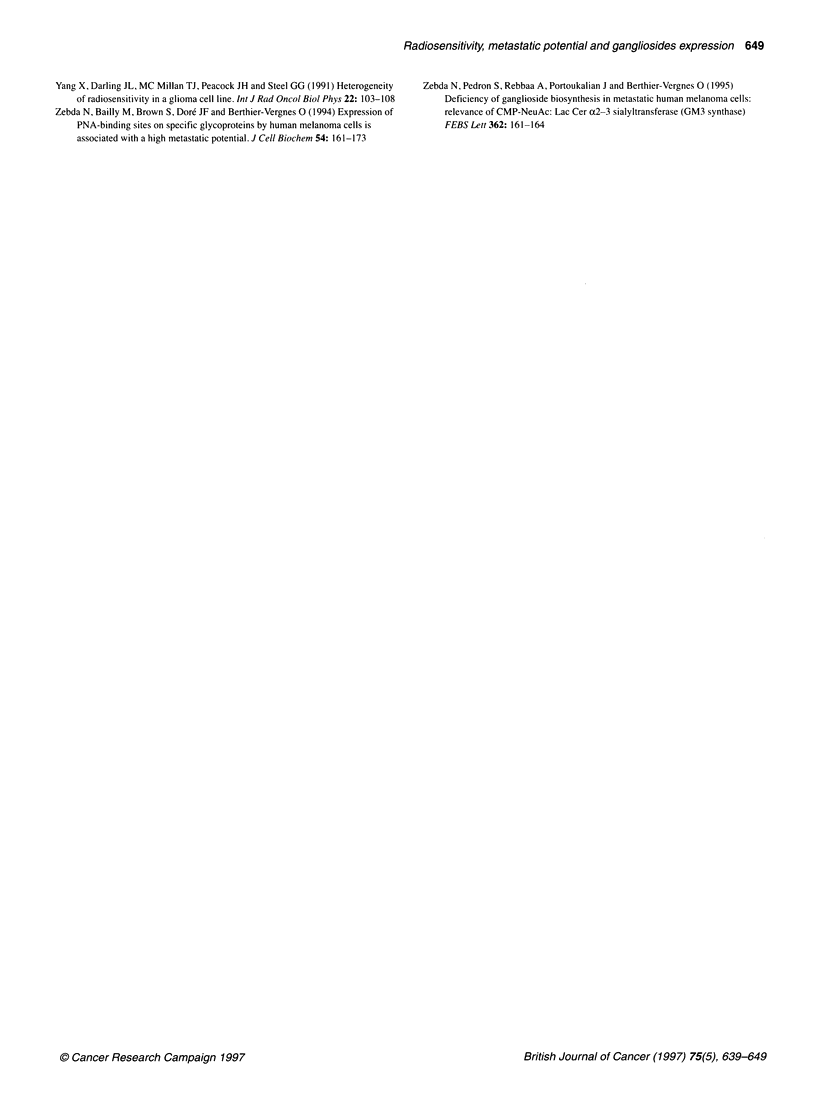# The gangliosides as a possible molecular coupling factor between the proportion of radiosensitive cells in vitro and the metastatic potential in vivo within a human melanoma cell line.

**DOI:** 10.1038/bjc.1997.115

**Published:** 1997

**Authors:** C. P. Thomas, A. Buronfosse, J. Portoukalian, B. Fertil

**Affiliations:** The Institut National de la Santé et de la Recherche Médicale (INSERM), Laboratoire d'Immunologie et de Cancérologie Expérimentale (Unité INSERM ex218), Lyon, France.

## Abstract

With an experimental model of spontaneous lung metastases in immunosuppressed newborn rats, seven clones and variants with different metastatic potential and gangliosides expression were derived from a single parental human melanoma cell line M4Be. The cellular radiosensitivity of M4Be and its seven sublines was estimated using an in vitro colony assay. The total amount of gangliosides in M4Be and its seven sublines was determined by cell extraction and thin-layer chromatography, while the expression of GD3 gangliosides was estimated by flow cytometry with a monoclonal antibody. The radiation-cell survival curves of most clones and variants derived from M4Be showed a zero dose extrapolation clearly lower than 100%, suggesting that two populations of cells of very different radiosensitivity coexist within each of these clones and variants. Although the proportion of radiosensitive cells could be estimated from the shape of the survival curve, its radiosensitivity is too high to be properly evaluated by the colony assay. The eight survival curves differ essentially in the proportion of radiosensitive cells--which varied from 0% to 40% among M4Be and its seven sublines--whereas the cellular radiosensitivity of the radioresistant population was similar among them. The metastatic potential in vivo of M4Be and its seven sublines was not significantly related to the cellular radiosensitivity of their corresponding radioresistant population, but significantly increased with the fraction of radiosensitive cells. This relationship is valid only when the highly metastatic cells are cultured for no more than five passages in vitro as the fraction of radiosensitive cells is rapidly lost during subcultures. The relationship remains valid in vivo as metastatic melanoma-bearing newborn rats whole body irradiated with 20 cGy show no lung metastasis compared with controls. The radiosensitive cell fraction is inversely correlated with both the total ganglioside content (r = 0.84, P < 0.02) and the number of cells positively labelled with the monoclonal antibody directed to GD3 (r = 0.92, P < 0.001). The incubation of a radiosensitive clone with the exogenous bovine brain ganglioside GM1 significantly increases the proportion of radioresistant cells and suppresses its metastatic potential, while the inhibition of the endogenous gangliosides synthesis in the radioresistant cell line M4Be increases the proportion of radiosensitive cells. This study provides a possible explanation for the correlation between the metastatic potential and the proportion of radiosensitive cells within the seven sublines derived from a single parental human melanoma cell line.


British Journal of Cancer (1997) 75(5), 639–649
© 1997 Cancer Research Campaign

### The gangliosides as a possible molecular coupling factor between the proportion of radiosensitive cells in vitro and the metastatic potential in vivo within a human melanoma cell line

**CP Thomas[1], A Buronfosse[1], J Portoukalian[1] and B Fertil[2]**

[1]The Institut National de la Santé et de la Recherche Médicale (INSERM), Laboratoire d'Immunologie et de Cancérologie Expérimentale (Unité INSERM ex218), Centre Léon Bérard, 69008 Lyon, France; [2]Laboratoire d'Imagerie Biomédicale (Unité INSERM 66), CHU Pitié-Salpétrière, Paris, France

**Summary** With an experimental model of spontaneous lung metastases in immunosuppressed newborn rats, seven clones and variants with different metastatic potential and gangliosides expression were derived from a single parental human melanoma cell line M4Be. The cellular radiosensitivity of M4Be and its seven sublines was estimated using an in vitro colony assay. The total amount of gangliosides in M4Be and its seven sublines was determined by cell extraction and thin-layer chromatography, while the expression of GD3 gangliosides was estimated by flow cytometry with a monoclonal antibody. The radiation–cell survival curves of most clones and variants derived from M4Be showed a zero dose extrapolation clearly lower than 100%, suggesting that two populations of cells of very different radiosensitivity coexist within each of these clones and variants. Although the proportion of radiosensitive cells could be estimated from the shape of the survival curve, its radiosensitivity is too high to be properly evaluated by the colony assay. The eight survival curves differ essentially in the proportion of radiosensitive cells – which varied from 0% to 40% among M4Be and its seven sublines – whereas the cellular radiosensitivity of the radioresistant population was similar among them. The metastatic potential in vivo of M4Be and its seven sublines was not significantly related to the cellular radiosensitivity of their corresponding radioresistant population, but significantly increased with the fraction of radiosensitive cells. This relationship is valid only when the highly metastatic cells are cultured for no more than five passages in vitro as the fraction of radiosensitive cells is rapidly lost during subcultures. The relationship remains valid in vivo as metastatic melanoma-bearing newborn rats whole body irradiated with 20 cGy show no lung metastasis compared with controls. The radiosensitive cell fraction is inversely correlated with both the total ganglioside content ($r = 0.84$, $P < 0.02$) and the number of cells positively labelled with the monoclonal antibody directed to GD3 ($r = 0.92$, $P < 0.001$). The incubation of a radiosensitive clone with the exogenous bovine brain ganglioside GM1 significantly increases the proportion of radioresistant cells and suppresses its metastatic potential, while the inhibition of the endogenous gangliosides synthesis in the radioresistant cell line M4Be increases the proportion of radiosensitive cells. This study provides a possible explanation for the correlation between the metastatic potential and the proportion of radiosensitive cells within the seven sublines derived from a single parental human melanoma cell line.

**Keywords:** human melanoma clone; spontaneous metastatic potential in vivo; radiosensitivity in vitro; biphasic survival curve; proportion of radiosensitive cells in vitro; gangliosides

At the time of the primary tumour diagnosis, up to 60% of patients may have microscopic and/or clinically evident metastases (Liotta, 1990). The efficacy of radiotherapy of metastases is size dependent i.e. the smaller the tumour size, the higher the radiocurability; this relationship has been demonstrated both in clinical studies (Fletcher, 1963) and in experimental studies (Courdi and Malaise, 1980). However, tumours, even small ones, are heterogeneous with regard to radiosensitivity in vitro as radioresistant and radiosensitive clonal populations are coexisting in the tumour cell lines (Alexander, 1961; Gridna et al, 1975; Hill et al, 1979; Leith et al, 1982; Welch et al, 1983; Jenkins et al, 1986; Yang et al, 1991; Buronfosse et al, 1994; Thomas et al, 1995*a*); this feature may be unstable and could change with culture passages in vitro either towards more radioresistant cells, as observed with some murine melanoma clones (Hill et al, 1979) and the MTLn3 clone isolated from the rat mammary adenocarcinoma 13762NF (Welch et al, 1983), or towards more radiosensitive cells, as observed with the MTC clone isolated from 13762NF (Welch et al, 1983). The fact that tumours contain subpopulations of cells of different metastatic potential has also been observed (Fidler, 1973), although these subpopulations change in their metastatic properties with passages in vitro (Neri and Nicolson, 1981). It is likely that, in tumours, the fraction of radioresistant cells together with the ability of tumours to generate metastases are major factors of failure in radiotherapy. As ionizing radiation is a strong mutagenic agent, it is believed that radiotherapy may allow the radioresistant tumour cells to make their last mutation during treatment, hence leading to the development of metastases. As far as we are aware, this concept

*Received 4 April 1996*
*Revised 29 August 1996*
*Accepted 4 September 1996*

*Correspondence to:* CP Thomas, INSERM, Laboratoire d'Histologie-Embryologie, Faculté de Médecine de Rangueil, 133 route de Narbonne, 31062 Toulouse Cedex, France

Parts presented as posters at the 42nd and 43rd Annual Meetings of the Radiation Research Society, Nashville, USA, 29 April–4 May 1994, and San Jose, USA, 1–6 April 1995.

**Table 1** The total ganglioside content, the number of cells positively labelled with the monoclonal antibody directed to the surface disialoganglioside GD3, the plating efficiency in vitro and the spontaneous metastatic potential in the immunosuppressed newborn rats of the human melanoma cell line M4Be and the seven sublines derived from M4Be

| Cells | Total gangliosides[a] | Number of cells GD3+[b](± s.e.) (%) | Plating efficiency in vitro (± s.d.) (%) | Metastatic incidence[c] (%) | Median lung nodules per rat (range) | Metastatic potential |
|---|---|---|---|---|---|---|
| Parental | 0.943 | 40 (± 8) | 74 (± 6) | 36 (5/14) | 0 (0–30) | Low |
| Clone 1 | 1.511 | 41 (± 8) | 42 (± 13) | 12 (4/34) | 0 (0–100) | Low |
| Clone 2 | 1.148 | 28 (± 6) | 54 (±10) | 83 (10/12) | 8[d] (0–180) | Medium |
| Clone 3 | 0.924 | 20 (± 5) | 46 (± 4) | 67 (8/12) | 53[d] (0–200) | Medium |
| Subvariant 1– | 0.883 | 28 (± 6) | 40 (± 8) | 100 (12/12) | 97[d] (46–>>300) | Medium |
| Variant 1 | ND | 17 (± 4) | 33 (± 8) | 100 (21/21) | >250[e] (2–>>300) | High |
| Subvariant 1+ | 0.607 | 15 (± 1) | 34 (± 5) | 100 (12/12) | >250[e] (3–>250) | High |
| Clone 4 | 0.322 | 2 (± 0.3) | 25 (± 13) | 100 (12/12) | 200[e] (28–>>300) | High |

[a]Expressed in μg of sialic acid mg$^{-1}$ protein. Data from Thomas et al (1996). [b]Data from Thomas et al (1995*b*). [c]The number of rats with lung metastasis/the number of rats injected (reported from Thomas et al 1995*a*). [d]The median is significantly higher than that obtained with clone 1 ($P$ <0.05). [e]The median is significantly higher than that obtained with subvariant 1– ($P$<0.05). Statistical analysis was done using the non-parametric Wilcoxon test. ND, not determined.

has never been verified experimentally and no relationship has been established so far between the metastatic potential of tumour cells in vivo and their radioresistance in vitro (see review by Suit et al, 1994). However, this relationship was mainly sought among tumour cell lines. Within a given tumour cell line, in contrast, we have observed recently that these two independent biological parameters appear to be correlated provided that the highly metastatic cells are cultured for no more than five passages in vitro (Thomas et al, 1995*a*). We observed that the higher the metastatic potential of the seven sublines derived from a single parental human melanoma cell line (M4Be), the higher their cellular radiosensitivity, as estimated using an in vitro colony assay.

To find a possible explanation for this relationship, we have characterized the sublines derived from M4Be by their surface gangliosides expression (ganglioside is the trivial name for sialic acid-bearing glycolipids). Indeed, the data in the literature suggest that, for the highly radiosensitive cells, molecular changes in the plasma membrane could be involved in ionizing radiation-induced cell death (Ramakrishnan et al, 1993) and that surface glycolipids may be involved (Kono et al, 1990; Haimovitz-Friedman et al, 1994). The expression of these surface molecules changes dramatically in many cells during the oncogenic transformation, suggesting a specific role for membrane glycolipids in the regulation of cell growth and cellular interaction (Hakomori, 1981). Although the gangliosides are ubiquitous plasma membrane molecules of essentially all the eukaryotic cells, the research of their biological functions is a field still in its infancy. Gangliosides which are shed by tumour cells into the serum may suppress the cellular immune response in vivo, thereby facilitating the tumour progression (Li et al, 1995). The results from our laboratory suggest that the deficiency of the gangliosides synthesis at the cell surface of some sublines derived from the M4Be human melanoma cell line is associated with both a high spontaneous metastatic potential in vivo (Thomas et al, 1995*b;* Zebda et al, 1995) and high radiosensitivity in vitro (Thomas et al, 1996). This study provides evidence that the gangliosides may be a possible molecular factor linking the proportion of radiosensitive cells detected in some sublines of the human melanoma cell line M4Be to their metastatic potential in vivo.

### MATERIALS AND METHODS

### Human melanoma cells

The clone IC8 was selected from the human-characterized melanoma cell line M4Be that was established from a patient's lymph node melanoma metastasis (Jacubovich and Doré, 1979). The selection and cloning methods have been described previously (Bailly and Doré, 1991). The variant T1p26 was established in culture after two direct successive transplantations of the M4Be tumours into immunosuppressed newborn rats (Bailly et al, 1993). The variant T1p26 is itself heterogeneous, containing two populations of cells that were separated by flow cytometry for their ability to bind the PNA lectin (peanut agglutinin lectin); the PNA recognizes specific glycoproteins at the cell surface, and the binding is higher for the subvariant T1p26R than the subvariant T1p26L; the metastatic potential of the subvariant T1p26R is significantly higher than that of the subvariant T1p26L (Zebda et al, 1994; Table 1). The clones T1C11, T1C6 and T1C3 were obtained by limiting dilution from the T1p26 variant. With the exception of the T1C6 and T1C11 clones, the derivation of the variants and clones from the M4Be cell line has been previously described (Zebda et al, 1994). The common origin of all these cell sublines is attested by their karyotype and by their sharing common marker chromosomes with the parental cell line M4Be; the cells appeared hypertriploid, showing quite similar modal

© Cancer Research Campaign 1997

numbers (around 70) (Bailly and Doré, 1992). For sake of clarity, they are referred to below as clone 1 (IC8), clone 2 (T1C11), clone 3 (T1C6), clone 4 (T1C3), variant 1 (T1p26), subvariant 1– (T1p26L), subvariant 1+ (T1p26R) and M4Be (parental cell line). The cell cultures obtained from the seven clones and variants were frozen a few passages after their isolation.

### Lung spontaneous metastasis assay

The M4Be cell line and the seven clones and variants have all been characterized for their ability to give low, intermediate and high numbers of spontaneous metastases in the lungs of the immunosuppressed newborn rats (Table 1). The lung metastatic potential was measured using a standardized protocol previously described (Bailly et al, 1991). Briefly, the cells taken from the frozen stock were cultured for at least one passage and, at day 0, Wistar rats not older than 24 h were injected subcutaneously (SC) with $10^6$ cells in 0.1 ml of phosphate-buffered saline (PBS) in the thorax area, together with an optimal dose of anti-thymocyte serum (ATS) in the dorsum (0.05 ml). The ATS injection was repeated on days 2, 7 and 14. All the sublines derived from M4Be, whatever their metastatic potential, produced SC tumours that were growing at the injection site. The animals were sacrificed on day 21, and the number of rats with lung metastasis *vs* the number of rats injected was determined. The number of lung nodules was scored, and the median lung nodules per rat was calculated. The median was chosen to take into account the total number of rats injected, even those without lung metastases as it was observed that in some cases, the probability for a rat to have no metastasis was not negligible (Table 1). The absence of occult metastases in the lungs with no visible metastases was confirmed by a histological examination. All experiments were performed according to the national regulations for animal welfare.

### Expression of the cell surface gangliosides

The total ganglioside content in the M4Be cell line and its seven sublines was determined by cell extraction and thin layer chromatography as previously described (Thomas et al, 1996). The expression of the disialoganglioside GD3 in the M4Be melanoma cell line and its seven sublines were determined by flow cytometry using a method already published (Thomas et al, 1995*b*, 1996). Briefly, after 4 days of culture, the early confluent cells were detached with 0.02% EDTA; the number of cells GD3 positive in $10^6$ cells was detected by immunofluorescence, using a primary murine monoclonal antibody 4F6 (IgG3) directed to GD3 (45 min of incubation at ambient temperature) and a mouse total immunoglobulin (second antibody) coupled to fluroscein (30 min of incubation at ambient temperature). The cells were rinsed with PBS after the incubation with the primary antibody and the secondary antibody coupled to fluoroscein. The antibody 4F6 was produced after immunization of the Balb/C mice with GD3 purified from the human melanoma tumours and fusion with the SP2 mouse myeloma. As shown by ELISA (Portoukalian et al, 1991) and TLC immunostaining (Portoukalian and Bouchon, 1986), the antibody reacts with GD3, *O*-acetyl GD3 and GT1a but not with GM3, GD2 and GT3 (C Pinatel and J Portoukalian, unpublished results). A flow cytometer FACScan was used to compare the number of fluorescent cells detected in PBS with no primary 4F6 antibody and the second antibody coupled to fluoroscein (control) to the number of fluorescent cells detected in the presence of the primary 4F6 antibody and the second antibody coupled to fluoroscein. Two thousand cells were analysed.

### Irradiation and the cell radiosensitivity measurement

The radiosensitivity experiments were performed with the cells obtained from the frozen stock; the cells were not cultured more than 3 months after the cell defrost, i.e. no more than 15 passages. The cells were cultured as monolayers in plastic flasks (Corning) and maintained at 37°C in an atmosphere of 5% carbon dioxide in air. The cells were grown in McCoy 5A medium (Gibco), supplemented with 10% decomplemented fetal calf serum (Gibco), 1% antibiotics (penicillin, streptomycin), 1% Hepes 1M and 1% glutamine. The cultures were routinely checked and found free of mycoplasma. In the growth phase, the doubling time of the clones and variants were about 24 h without any apparent relationship with the metastatic ability (Bailly et al, 1993). The plating efficiencies of clones and variants are reported in Table 1. The clones 1 and 4, the variants 1 and the subvariants 1– and 1+ were irradiated with γ-rays from a $^{60}$Co source that provided a mean dose rate of 0.58 ± 0.01 Gy $min^{-1}$ to the culture flasks. The clones 2 and 3 as well as the M4Be cell line were irradiated with high energy X-rays produced from a 5-MV linear accelerator that provided a mean dose rate of 4 Gy $min^{-1}$ to the culture flasks. The dose rates were checked with an ionization chamber, and the homogeneity of the dose over the irradiation field was verified with radiographic film (± 3%). Although the source of ionizing radiation and the dose rate was changed during the course of these experiments, no effect on the radiosensitivity is expected. Indeed, no significant differences were found in the radiosensitivity of human cell lines for dose rates in this range [e.g. for Hela cells with dose rate varying from 7 Gy $min^{-1}$ to 0.45 Gy $min^{-1}$ (Hall, 1972) and for five fibroblasts with dose rate varying from 1 Gy $min^{-1}$ to 0.5 Gy $min^{-1}$ (Badie et al, 1996)]. The experiments were carried out with the early confluent cultures, i.e. at a time when about 80% of the cells are blocked in the $G_1$ phase. The cells were detached with 0.05% trypsin–0.02% EDTA in PBS, and the single cell suspensions were seeded on 25 $cm^2$ plastic flasks (Corning) at cell numbers appropriate for the colony formation. Two flasks were used at each radiation dose and the number of radiation doses was eleven varying from 5 cGy to 7 Gy. Twenty-four hours after plating, the cultures were irradiated after the electronic equilibrium and at room temperature under aerobic conditions. In our experimental conditions, i.e. cells plated at low density and irradiated 24 h after plating, the influence of cell multiplicity (the number of cells per potential colony-forming unit) on the calculation of cellular radiosensitivity is negligible. After 2 weeks of incubation without changing the medium in an atmosphere of 5% carbon dioxide in air, the colonies were rinsed with PBS, fixed and stained with a mixture of alcohol 20%–crystal violet (1:1, by volume). The colonies containing more than 50 cells were scored, and the cell survival curves were established. The surviving fraction S(D), for a dose D, was calculated as the plating efficiency of the irradiated cells over that of the unirradiated cells; the plating efficiency is the number of colonies counted over the number of cells inoculated at day 0.

### Analysis of survival curves

### *The model*

The shapes of most of the survival curves of the clones and variants derived from the human melanoma cell line M4Be clearly suggest the existence of two populations of cells with very different radiosensitivity. The survival curves are adequately described by a sum of linear-quadratic terms, an extra parameter (λ) being

© Cancer Research Campaign 1997

**Figure 1** The inter-experiments variation of the survival curves of clone 4 with passages after the cell defrost. Five experiments are represented: ●, passage 1; ◆, passage 4; ◇, passage 8; ○, passage 11; and ■ passage 15. The experiments were carried out with the in vitro clonogenic assay

**Figure 2** The variations of both the proportion of radioresistant cells (λ, **A** and the mean inactivation dose of the radioresistant population ($\bar{D}_R$, **B**) along with the number of passages after the cell defrost of the seven clones and variants derived from the parental human melanoma cell line M4Be. The estimated 95% confidence intervals obtained for clone 4 are shown (typical experiment, reproduced three times). The experiments were carried out with the in vitro clonogenic assay–

introduced to evaluate the relative proportion of these two populations. The following equation was subsequently proposed:

$$S = \lambda e^{-\alpha_R D - \beta_R D^2} + (1 - \lambda) e^{-\alpha_S D - \beta_S D^2} \quad (1)$$

where $\alpha_R \beta_R$ and $\alpha_S \beta_S$ are the parameters of the radiosensitivity of the two radioresistant and radiosensitive populations respectively; λ is the proportion of radioresistant cells. This model was found to be satisfactory, but the radiosensitivity of the radiosensitive population is too high to be properly estimated with the in vitro colony assay, and insufficient data were available in the very low dose range (i.e. lower than 0.12 Gy). The model was therefore simplified:

$$S = \lambda e^{-\alpha_R D - \beta_R D^2} \quad (2)$$

The mean inactivation dose, equal to the area under the survival curve plotted in linear coordinates, is used as an index of the radiosensitivity and calculated by the numerical integration of $S(D)$. The radiosensitivity of the whole population, as well as that of the resistant part, was evaluated following the method proposed by Fertil et al (1984). The mean inactivation dose of the total population ($\bar{D}_T$) was calculated using equation 2, whereas the mean inactivation dose of the radioresistant population ($\bar{D}_R$) was calculated using the same equation with λ = 1.

### *Statistical analysis*

The relationship between the proportion of radioresistant cells (λ) and the metastatic potential was tested using the analysis of variance (ANOVA).

### RESULTS AND DISCUSSION

### At the clonal level, most of the radiation survival curves of melanoma cells are biphasic in nature

As an example, it can be seen that four out of five of the cell-survival curves obtained with clone 4 do not extrapolate to one (Figure 1). This behaviour, which is observed for most of the clones and variants, strongly suggests the existence of two populations with very different radiosensitivity within each clone and variant. The dose range investigated does not allow the evaluation of the radiosensitivity of the radiosensitive population. Although, since the pioneering work of Puck and Marcus (1956), most of the in vitro human cell-survival curves described in the literature do not display such a feature, several multiphasic curves have already been established with mammary adenocarcinoma clones (Welch et al, 1983), variants of CHO cells (Denekamp et al, 1989), fibroblasts from skin biopsies (e.g. Loeffler et al, 1990), a glioblastoma cell line (Allalunis-Turner et al, 1993), a soft-tissue sarcoma cell line (Dahlberg et al, 1993), lymphocytes from the blood of healthy volunteers (Uzawa et al, 1994) and endometrial cancer cell lines (Rantanen et al, 1995). Apoptosis has been proposed as a possible mechanism in producing the high radiosensitivity of the primary human uroepithelium cells at low radiation doses of Cobalt-60 gamma rays (Mothersill et al, 1995).

### The proportion of radiosensitive cells in the metastatic clones is reduced rapidly during the in vitro subcultures

Cell survival curves from the seven clones and variants derived from the parental cell line M4Be were drawn from experiments

 © Cancer Research Campaign 1997

**Figure 3** **(A)** The cell-survival curves of clone 1 (○), clone 4 (●) and the M4Be parental cell line (+) (M4Be data from one experiment). **(B)** The cell-survival curves of clone 2 (○) and clone 3 (●). The survival curves of clones 1 and 4 are represented for the comparison (dashed lines). **(C)** The cell-survival curves of variant 1 (●) and its two cell populations: subvariants 1+ (□) and 1– (◇) which were selected for their high and low PNA-binding ability respectively. The survival curves of clones 1 and 4 are represented for comparison (dashed lines). Pooled data from two experiments (17–22 points per clones and variants) were adequately fitted, using the linear–quadratic model, if an extra parameter (λ) was introduced to account for a zero-dose extrapolation often found to be different from one (see Material and methods). The experiments were carried out with the in vitro clonogenic assay carried out at different passages after the cell defrost. Systematic interexperimental variations could be detected (Figure 1). It seems that, for some sublines, the radioresistance increases during the culture passage in vitro. Such a drift has already been reported with murine melanoma clones (Hill et al, 1979) and a rat mammary adenocarcinoma clone MTLn3 (Welch et al, 1983). To

**Table 2** The cell survival curve parameters of the seven sublines derived from the parental cell line M4Be. The data were obtained with the cells cultured for no more than five passages in vitro after the cell defrost (two experiments were pooled – 17 to 22 points per subline)

| Cells | $\bar{D}_T$ (Gy)[a] ($\bar{D}_T = \lambda \times \bar{D}_R$) | λ[b] (95% CI) | $\bar{D}_R$ (Gy)[c] |
|---|---|---|---|
| M4Be[d] (parental line) | 3.18 | 1.10 (1.01–1.19) | 2.89 |
| Clone 1 | 3.05 | 1.09 (0.71–1.63) | 2.80 |
| Clone 2 | 2.63 | 0.93[e] (0.86–1.00) | 2.89 |
| Clone 3 | 2.53 | 0.91[e] (0.78–1.05) | 2.72 |
| Subvariant 1– | 2.94 | 0.98[e] (0.84–1.15) | 3.00 |
| Variant 1 | 2.41 | 0.82[e] (0.74–0.91) | 2.94 |
| Subvariant 1+ | 1.69 | 0.69[e] (0.48–0.98) | 2.45 |
| Clone 4 | 1.37 | 0.59[e] (0.41–0.82) | 2.32 |
| Mean (CV) | 2.48 (26%) | 0.89 (20%) | 2.75 (9%) |

[a]The mean inactivation dose of the whole population. [b]The proportion of radioresistant cells and estimated 95% confidence intervals. [c]The mean inactivation dose of the radioresistant population. [d]One experiment only (11 points). [e]Statistically different from the parental cell line M4Be ($P < 0.05$) CV, coefficient of variation.

investigate this phenomenon, radiosensitivity parameters were evaluated for each experiment. However, important variations were expected as three parameters had to be estimated from a small set of data. For example, coefficients of variation of 34%, 26% and 42% for the mean inactivation dose of the total population ($\bar{D}_T$), the mean inactivation dose of the radioresistant population ($\bar{D}_R$) and the proportion of radioresistant cells (λ), respectively, were obtained with the five separate experiments from clone 4 (Figure 1). The parameters λ and $\bar{D}_R$ were plotted against the number of passages after the cell defrost for the seven clones and variants (Figure 2). The drift of λ toward unity is particularly obvious for the highly metastatic cells (clone 4, variant 1 and subvariant 1+), whereas λ appeared relatively stable and close to one for the cells with low metastatic potential (clone 1) and intermediate metastatic potential (clones 2 and 3, subvariant 1–). Indeed, during the 15 passages undergone by clone 4 in the culture after the cell defrost, λ varied from 27% to 100% while it remained around 100% in clone 1 during the 10 passages (Figure 2A). In contrast, during the in vitro subcultures after the cell defrost, no systematic variation in the radiosensitivity of the radioresistant population, as estimated from $\bar{D}_R$ could be detected (Figure 2B). To obtain an acceptable estimation of the radiosensitivity after the cell defrost, only the data measured after no more than five passages in vitro (two experiments) were used for establishment of the survival curve.

### The survival curves vary from one subline to another

The survival data obtained with clones 1 and 4 are presented in Figure 3A. Clone 4 is much more radiosensitive than clone 1, and the radiosensitivity of the parental cell line M4Be is similar to that obtained with clone 1 (Figure 3A). Clones 2 and 3 showed an intermediate response to radiation compared with that observed

© Cancer Research Campaign 1997

**Figure 4** The variations of both the proportion of radioresistant cells in vitro (λ) and the mean inactivation dose of the radioresistant population ($\bar{D}_R$) with the metastatic potential in vivo of the seven clones and variants derived from the human melanoma cell line M4Be. The 95% confidence intervals are represented. A significant correlation exists between λ and the metastatic potential: the higher the value of λ, the lower the metastatic potential ($P < 0.006$, ANOVA analysis)

with M4Be and clone 4 (Figure 3B). Figure 3C shows the survival curves obtained from variant 1 and the two subvariants 1+ and 1–; the two subvariants were separated by flow cytometry for their ability to bind PNA, a lectin recognizing specific glycoproteins at the cell surface (Zebda et al, 1994). The subvariant 1+, which binds more PNA than the subvariant 1–, is much more radiosensitive, while an intermediate response to radiation is observed for variant 1. Examination of the parameter values of the eight cell-survival curves ($\bar{D}_T$ and λ) confirms that the clones and variants derived from the M4Be parental cell line differ in their radiosensitivity (Table 2). Clone 1, subvariant 1– and the M4Be cell line are the most radioresistant; clone 4 and subvariant 1+ are the most radiosensitive, while clones 2 and 3 and variant 1 show an intermediate response to radiation.

This result is similar to previous observations showing the clonal variation in the radiosensitivity inside tumour cell lines obtained from lymphoma (Alexander, 1961), fibrosarcoma (Grdina et al, 1975), melanomas (Hill et al, 1979; Thomas et al, 1995*a*), the stomach (Jenkins et al, 1986), the colon (Leith et al, 1982), the breast (Dewyngaert et al, 1981; Welch et al, 1983) and glioblastomas (Yang et al, 1991; Buronfosse et al, 1994).

### The survival curves of the seven clones and variants differ essentially by the proportion of radioresistant cells

The proportions of radioresistant cells (λ) were: 1.1, 1.09 and 0.98, respectively, for the M4Be parental cell line, clone 1 and subvariant 1–; 0.93, 0.91 and 0.82, respectively, for clones 2, 3 and variant 1; 0.69 and 0.59, respectively, for subvariant 1+ and clone 4. This variation is characterized by a coefficient of variation (CV) of 20% (Table 2). In contrast, in the M4Be cell line and the seven sublines derived from M4Be, the variation of the radiosensitivity of the radioresistant population as measured by $\bar{D}_R$ can be considered as non-significant, being typical of results based only on two experiments (CV = 9%, Table 2).

Although the proportion of radiosensitive cells is known (1–λ), their radiosensitivity is too high to be properly estimated using the in vitro colony assay. However, considering the survival at the first dose investigated, the mean inactivation dose of the radiosensitive population ($\bar{D}_S$) could not be higher than 0.2 Gy. This observation is consistent with the hypersensitivity that was observed in the initial part of the survival curve of a human tumour cell line; depending on the model used to analyse the data, $\bar{D}_S$ varied between 0.9 Gy and 0.04 Gy (Lambin et al, 1993). Thus, the variation in the radiosensitivity of the seven clones and variants derived from the M4Be melanoma cell line essentially results from difference in the proportion of radioresistant cells. It should be pointed out that a similar behaviour has already been described for the variants of CHO normal cells (Denekamp et al, 1989). Using a similar survival-curve model, they found in several cell variants a fraction of radioresistant cells varying from 0.4% to 12% of the total population, and hence these authors could state that 'the reported difference (in their radiosensitivity) results from the variability in the fraction of cells expressing radioresistance'.

### The metastatic potential of the human melanoma sublines is related to their radiosensitive cell fraction but not to the radiosensitivity of their radioresistant cell fraction

As reported in Table 1, the seven clones and variants derived from M4Be have different metastatic capacity. Clone 1 and the M4Be cell line have the lowest metastatic potential whereas clone 4, variant 1 and subvariant 1+ are highly metastatic. Clones 2 and 3 and subvariant 1– have an intermediate metastatic capacity. The radiosensitivity in vitro of these clones and variants is related to their metastatic capacity in vivo (Table 1 and 2). More precisely, the proportion of radioresistant cells (λ) in each subline is significantly related to their metastatic potential: the lower λ is, the higher the metastatic potential ($P < 0.006$) (Figure 4A). This relationship is valid only as long as the radiation survival curves of the highly metastatic cells are biphasic, i.e. when these cells are cultured for no more than five passages. With additional passages in vitro (up to 15), the survival curves of the highly metastatic cells become monophasic and, as the proportion of radioresistant cells increases (Figure 2A), the relationship with the metastatic potential loses its significance. Furthermore, when the radiosensitivity of the radioresistant clonal fraction (as estimated by the $\bar{D}_R$ values in Table 2) is taken into account, there is no significant relationship with the metastatic potential given in Table 1 ($P = 0.3$, Figure 4B). Moreover, the radiosensitivity of the radioresistant clonal fraction does not change with the passage level (see Figure 2).

© Cancer Research Campaign 1997

**Table 3** The metastatic potential of clone 4 cells that have received a priming dose of 2 Gy was compared with that of the control clone 4 cells that have not received the priming dose. In addition, the radiosensitivity of these cells was compared. The metastatic potential and radiosensitivity experiments were performed 24 h after the priming dose of 2 Gy. The cells have been cultured for no more than five passages before injection to the newborn rats and the passage number after cell defrost was the same for the control and the experimental group.

| | | Metastatic potential | | | Radiosensitivity parameters | | |
|---|---|---|---|---|---|---|---|
| | Number of tumour cells injected subcutaneously | Incidence[a] | Average number of lung nodules in rats with metastasis (± s.d.) | Lung invasion efficiency[b] (± s.d.) (%) | $\bar{D}_T$[c] (Gy) | $\bar{D}_R$[d] (Gy) | λ[e] (95% CI) |
| Control cells | $10^6$ | 4/10 (40 %) | 200 (± 108) | 0.1 (± 0.06) | 2.07 | 2.38 | 0.87 (0.77–0.98) |
| Cells preirradiated with 2 Gy[f] | $2.2 \times 10^6$ | 5/12 (42 %) | 83 (± 111) | 0.02[g] (± 0.03) | 2.51 | 2.38 | 1.05 (0.97–1.14) |

[a]The number of rats with lung metastasis/the number of rats injected. [b]Number of lung nodules / (number of tumour cells injected subcutaneously × plating efficiency). The plating efficiency was 20% for the control cells and 19% for the cells that received a priming dose of 2 Gy. [c]The mean inactivation dose of the whole population. [d]The mean inactivation dose of the radioresistant population. [e]The proportion of radioresistant cells and the 95% confidence intervals. [f]In this experimental group, the number of cells injected was multiplied by 2.2 compared with those in the control group to account for the 55% clonogenic cells death due to the preirradiation at 2 Gy (SF2 = 0.45). [g]$P$ = 0.035 compared with control cells ($t$-test).

As a working hypothesis, it was postulated that the metastatic potential of our clones and variants is a specific feature of their radiosensitive cell population. To test this hypothesis, both the radiosensitivity in vitro and the metastatic capacity of the clone 4 cells cultured for no more than five passages in vitro were measured after a priming dose of 2 Gy given 24 h before experiments – a procedure that should eliminate the radiosensitive cells. The results presented in Table 3 support the concept of the two populations, as the radiosensitivity of the cells that have received a priming dose of 2 Gy was lower than that obtained for the control cells that have not received the priming dose. Moreover, the metastatic potential in vivo of the cells that have received a priming dose of 2 Gy and injected subcutaneously 24 h after the irradiation in the immunosuppressed newborn rats is fivefold lower than that of the control cells that have not received the priming dose of 2 Gy ($P$ = 0.035, $t$-test) (Table 3). This result clearly shows a decrease of the metastatic potential upon the disappearance of the radiosensitive population. Moreover, the separation by flow cytometry of the two populations in variant 1, selected for their high and low PNA-binding ability, showed that the higher the proportion of radiosensitive cells in each subpopulation, the higher their metastatic potential (Tables 1 and 2 and Figure 3). We suggest therefore that the metastatic potential of the seven clones and variants derived from the M4Be cell line depends on the radiosensitive population. This proposal does not necessarily imply that metastases are more radiosensitive than the tumour of origin as the heterogeneity may quickly reappear in situ. Indeed, the characterized melanoma cells isolated from the lung metastasis were not significantly more radiosensitive than those isolated from the primary melanoma tumour (CP Thomas et al, unpublished results). Although the latter result is consistent with those obtained by Rofstad (1992), the data in this field are controversial. It was reported that small-size lung metastases irradiated in situ were more radiosensitive than the single cell suspensions obtained from the mammary tumour of origin and irradiated in vitro (Fu et al, 1976). In vitro cell survival curves of the lung metastases were also determined (Welch et al, 1983), and the survival curve obtained with a clone derived from an original subcutaneous mammary tumour was found to be triphasic. It can be noticed that the initial part of the latter survival curve, revealing a highly radiosensitive subpopulation of cells, is similar to the survival curve observed with the lung metastases. A similar behaviour regarding the radiosensitivity in vitro was detected recently with a human cell line established from an endometrial adenocarcinoma and a supraclavicular fossa metastasis originating from the same patient (Rantanen et al, 1995).

In the present study, provided that the cells were cultured for no more than five passages in vitro, a significant relationship was defined between the radiosensitive cell fraction of the sublines derived from a single parental melanoma cell line and their metastatic potential. The radiosensitive cell fraction, which is important in the highly metastatic cells, is however rapidly lost over the time in culture. The causes of such transcients in the radiosensitivity of the highly metastatic cells remains unknown to us. A similar variabilty is also encountered with the metastatic incidence of the highly metastatic cells which for example in clone 4 is found to vary from 100% (Table 1) to 40% (Table 3) even though the experiments were conducted in similar conditions, with the cells being cultured for a short period in vitro. This is however not surprising as many technical factors are known to infuence the metastatic incidence, such as the level of immunosuppression achieved by ATS, the method of cell injection, etc. (Bailly and Doré, 1991; Bailly et al, 1991). It is remarkable that, despite all these factors of variability, we have been able to observe some significant correlations.

Our results suggest that a whole-body irradiation of metastatic tumour-bearing animals with a low dose of irradiation should result in a significant decrease of the metastatic cell population. Experiments were carried out to test this hypothesis. The data in Table 4 show that, when metastatic-melanoma-bearing newborn rats are whole-body irradiated with a dose of 20 cGy immediately after the cell injection, a suppression of the metastatic potential of the tumour occurs. Consistently with our results, it was observed that the number of artificial lung metastases of a murine squamous cell carcinoma is reduced about twofold when the mice are whole-body irradiated, immediately after the tumour cell injection, with a

**Table 4** The spontaneous metastatic potential of clone 4 and the characterized melanoma cells obtained from the lung metastasis (Met cells)

| | Incidence[a] | Average number of lung nodules per rat (±s.d.) |
|---|---|---|
| Clone 4 cells (control) | 4/10 (40 %) | 200 (± 110) |
| Clone 4 cells (treated with GM1) | 0/10 | 0 |
| Met cells (control) | 3/15 (20 %) | 130 (± 60) |
| Met cells (WBI 20 cGy) | 0/10 | 0 |

The cells from the clone 4 were treated with the bovine brain monosialoganglioside GM1 (4 μM) 4 days in culture before their subcutaneous (s.c.) injection in the immunosuppressed newborn rats. The cells from the lung metastasis (Met) were s.c. injected in the immunosuppressed newborn rats that were whole-body irradiated (WBI) with 20 cGy immediately after the cell injection. The cells have been cultured for no more than five passages before injection to the newborn rats and the passage number after cell defrost was the same for the control and the experimental group. [a]The number of rats with lung metastasis over the number of rats injected.

dose of 20 cGy; although the effect was thought to result from a radiation-induced stimulation of the immune system (Hosoi and Sakamoto, 1993). As the results on the hyper-radiosensitivity of the highly metastatic cells were obtained using a clonogenic assay, with the cells plated at low density and irradiated 24 h later, this phenomenon would be relevant in a clinical situation under the same experimental conditions. For example, this may be applicable in a situation where the metastatic cells are isolated either after escaping from the primary tumour or as soon as they start growing in a distant tissue.

On the one hand, in a recent review of the data from six centres, no relationship was found between the metastatic potential of 222 human carcinoma biopsies, 24 experimental tumours of various histology, 21 tumor cell lines and 16 sublines and their radiosensitivity (Suit et al, 1994). On the other hand, in the present study within a single melanoma cell line, the metastatic potential of the clones and variants derived from the parental melanoma cell line was related to their proportion of radiosensitive cells, provided that the cells were cultured for no more than five passages in vitro after the defrost; this restrictive condition might account for the apparent discordance with previous studies as we have shown that the fraction of radiosensitive cells is progressively lost along with the passages in culture (Figure 2). If such a behaviour could be confirmed in other tumour cell lines, a unified view may be that the radiosensitivity of a given cell line containing only one major population (e.g. the parental M4Be cell line contains only a major radioresistant cell population) and the proportion of potentially radiosensitive cells within this cell line are independent parameters. This proportion of potentially radiosensitive cells, which is suggested to be responsible for the metastatic potential, varies at the clonal level within a given cell line and may vary from one cell line to another, whatever the radiosensitivity of the cell line. Thus, the radiosensitive cell lines may have a low metastatic potential, similar to the radioresistant ones, provided that the hyper-radiosensitive subpopulation is not present in these cell lines.

**Figure 5** The total gangliosides content (expressed in μg sialic acid $mg^{-1}$ protein) (**A**) and the proportion of cells positively labelled with the monoclonal antibody directed to the disialoganglioside GD3 (**B**) are inversely correlated to the proportion of radiosensitive cells in the human melanoma cell line M4Be and the seven sublines derived from M4Be ($r = 0.84$, $P < 0.02$ and $r = 0.92$, $P < 0.001$ respectively). The mean and the SEM of the data from four experiments are presented in **B**

### The deficiency in the surface gangliosides is a feature of the highly metastatic and highly radiosensitive human melanoma cells

The total gangliosides content and the number of cells positively labelled with the monoclonal antibody directed to the surface disialoganglioside GD3 in the early confluent cultures obtained from the human melanoma cell line M4Be and the seven sublines derived from M4Be is shown in Table 1. The lower the total amount of gangliosides, the higher the proportion of radiosensitive cells inside M4Be and its seven sublines (Figure 5). Also, the lower the number of GD3-positive cells in the early confluent cultures of M4Be and its seven sublines, the higher their proportion of radiosensitive cells (Figure 5). Similar results regarding the number of GD3+ cells were obtained with the exponentially growing cells. Thus, it seems unlikely that the number of cells GD3+ depends on the proliferation rate in non-synchronized cultures. We propose that, inside the human melanoma cell line M4Be, the cells deficient in gangliosides are more metastatic and more radiosensitive than those rich in gangliosides. This hypothesis is strengthened by the experiments which show that the radioresistance of the M4Be cells could be modified by changing their gangliosides status (Figure 6). Firstly, it was observed that blocking the biosynthesis of the gangliosides in the radioresistant cell line M4Be with the inhibitor Fumonisin B1 significantly reduces its proportion of radioresistant cells in vitro

© Cancer Research Campaign 1997

**Figure 6 (A)**. The effect of the endogenous gangliosides inhibitor Fumonisin B1 (6 days of incubation at 10μM in the culture medium) on the cellular radioresistance of the human melanoma cell line M4Be. The mean and the standard deviation from three experiments are represented for the control cells (■) and the treated cells (□). The data were adequately fitted using the linear–quadratic model if an extra parameter (λ) was introduced to account for a zero-dose extrapolation which was found to be significantly lower than 100% in the treated cells (see Materials and methods). (**B**). The radiation cell-survival curves of clone 4 treated or not with 4 μM exogenous bovine brain monosialoganglioside GM1 4 days before and during the irradiation. The mean and the standard deviation from two experiments are represented for the control cells (■) and the treated cells (□). The data were adequately fitted with the liner–quadratic model if an extra parameter (λ) is introduced to account for a zero-dose extrapolation which was significantly lower than 100% in the control cells (see Materials and methods)

(Figure 6A). Secondly, the incubation of the radiosensitive clone 4 with the exogeneous bovine brain monosialoganglioside GM1 (4 μM) for 4 days significantly increases the proportion of radioresistant cells in vitro (Figure 6B) and suppresses the metastatic potential in vivo (Table 4). The results are more extensively reported in two companion papers (Thomas et al, 1995*b*, 1996). Thus, the decrease of the gangliosides expression at the cell surface may be involved in the metastatic potential of human melanoma tumours.

This proposal is strengthened by the published data from our laboratory showing that, after the subcutaneous injection of cells from the human melanoma clone 1 (poorly metastatic and high gangliosides content in vitro) and clone 4 (highly metastatic and low gangliosides content in vitro) in newborn rats, the tumour cells proliferating at the injection site re-expressed the four common gangliosides of melanoma (GM3, GM2, GD3 and GD2), whereas the metastatic lung colonies of clone 1 and 4 were deficient in the gangliosides synthesis (Zebda et al, 1995). It seems that the cells with low gangliosides content inside these two sublines possess the highest ability to form lung metastases. Furthermore, our data show that the proportion of radioresistant cells (λ) in the highly metastatic human melanoma clones derived from a single poorly metastatic parental cell line M4Be increased rapidly during the time in culture (Figure 2). As we have established a correlation showing that the higher the value of λ, the higher the gangliosides content (particularly GD3) at the cell surface of sublines derived from M4Be (Figure 5), it is tempting to suggest that the increase of λ over time in culture in the highly metastatic clones is associated with a parallel increase in the gangliosides synthesis (particularly GD3). Although no systematic study was designed to verify this hypothesis, we have observed during the time course of our experiments that the proportion of cells which are GD3 positive in the radiosensitive and metastatic clone 4 could vary from 1% to 20% and that this change is roughly associated with an increase in λ. The reason(s) causing such transcients in the gangliosides expression at the surface of the radiosensitive and metastatic human melanoma cells is still unknown. The mechanism by which the change in the gangliosides status controlled the radiosensitive cell fraction and, hence, the metastatic potential is also unclear. We suggest that the electrostatic environment provided by the negatively charged sialic acid residues of the cell surface gangliosides may influence the radioresistant cell fraction as the cleaving with neuraminidase of more than 50% of these surface molecules significantly reduces λ (Thomas et al, 1996). These data support the postulate of Alper that, although DNA is usually considered as the main critical target for radiation, an additonal lethal effect may also be produced by the free-radicals that damage the cell membrane functions (Alper, 1979). We wish to emphasize that these membrane effects may occur at low doses of radiation in the hyper-radiosensitive cells.

In summary, the in vitro radiation cell-survival curves of the seven clones and variants derived from a human melanoma cell line differ essentially by the proportion of hyper-radiosensitive cells. This cell population is rapidly lost over the time in culture. Using $\bar{D}_R$ as an index of the radiosensitivity, it was found that the radiosensitivity of the radioresistant fraction of the cell population, which is not drifting with the passages, is similar among the seven sublines and the parental line. Therefore, $\bar{D}_R$ cannot be correlated with the metastatic potential. In the present study, a correlation was established between the metastatic potential and the hyper- radiosensitive fraction of the melanoma cells. Our data suggest that the metastatic potential of a cell line in vivo is due to the fraction of radiosensitive cells in vitro. A possible explanation for the correlation is provided in this study which shows that the change of the ganglioside status at the cell surface modifies the proportion of radiosensitive cells and hence, the metastatic potential of human melanoma.

### ACKNOWLEDGEMENTS

We would like to thank Christiane Bailly from the Department of Pathology for helping with the histopathology; Maryse Bailly from

© Cancer Research Campaign 1997

the Department of Molecular Oncology for providing her original data concerning the metastatic potential in vivo of clones 2 and 3; Noureddine Zebda for helping with the lung spontaneous metastasis assay; Valérie Combaret and Christiane Pinatel from the Department of Tumour Biology for helping respectively with the flow cytometer and the antibody 4F6 production; Chantal Ginestet, Claude Malet and Christian Mombard from the Department of Radiotherapy for helping with the irradiation and the dosimetry. This work was supported in part by a grant to CT from 'Association pour la Recherche sur le Cancer' (ARC) contract no. 2052.

### REFERENCES

Alexander P (1961) Mouse lymphoma cells with different radiosensitivities. *Nature* **192:** 572–573

Allalunis-Turner, MJ Barron, GM Day III, RS, Dobler KD and Mirzayans R (1993) Isolation of two cell lines from a human malignant glioma specimen differing in sensitivity to radiation and chemotherapeutic drugs. *Radiat Res* **134:** 349–354

Alper T (1979) *Cellular Radiobiology*. Cambridge University Press: Cambridge

Badie C, Alsbeih G, Reydellet I, Arlett C, Fertil B and Malaise EP (1996) Dose rate effect on the survival of irradiated hypersensitive and normal human fibroblasts. *Int J Radiat Biol* (in press)

Bailly M and Doré JF (1991) Human tumor spontaneous metastasis in immunosuppressed new born rats. II. Multiple selections of human melanoma metastatic clones and variants. *Int J Cancer* **49:** 750–757

Bailly M and Doré JF (1992) Clonal drift and role of chromosome dosage in human melanoma metastatic cell lines: a statistical analysis. *Anticancer Res* **12:** 1163–1172

Bailly M, Bertrand S and Dore JF (1991) Human tumor spontaneous metastasis in immunosuppressed new born rats. I. Characterization of the bioassay. *Int J Cancer* **49:** 457–466.

Bailly M, Bertrand S and Doré JF (1993) Increased spontaneous mutation rates and prevalence of karyotype abnormalities in highly metastatic human melanoma cell lines. *Melanoma Res* **3:** 51–61

Buronfosse A, Thomas CP, Ginestet C and Dorée JF (1994) Radiosensitivity *in vitro* of clonogenic and non-clonogenic glioblastoma cells obtained from a human brain tumor. *C R Acad Sci Paris* **317**: 1031–1041

Courdi A and malaise EP (1980) Effect of size of lymph node metastases on the radiation response: influence of misonidazole. *Radiat Res* **83:** 723–731

Dahlberg WK, Little JB, Fletcher JA, Suit HD and Okunieff P (1993) Radiosensitivity *in vitro* of human soft tissue sarcoma cell lines and skin fibroblasts derived from the same patients. *Int J Rad Biol* **63:** 191–198

Denekamp J, Whitmore GF and Jeggo P (1989) Biphasic survival curves for XRS radiosensitive cells: subpopulations or transient expression of repair competence? *Int J Rad Biol* **55:** 605–617

Dewyngaret JK, Leith JT, Peck RA, Bliven SF, Zeman EM, Marino SA and Glicksman AS (1981) Differential RBE values obtained for mammary adenocarcinoma tumor cell subpopulations after 14.8 Mev neutron irradiation. *Radiat Res* **88:** 118–131

Fertil B, Dertinger H, Courdi A and Malaise EP (1984) Mean inactivation dose: a useful concept for intercomparison of human cell survival curves. *Radiat Res* **99:** 73–84

Fidler IJ (1973) Selection of successive tumor cell lines for metastasis. *Nature* **242:** 148–149

Fletcher GH (1963) Clinical dose response curves of human malignant epithelial tumors. *Br J Radiol* **46:** 1–12

Fu KK, Phillips TL and Wharam MD (1976) Radiation response of artificial pulmonary metastases of the EMT6 tumor. *Int J Rad Oncol Biol Phys* **1:** 257–260

Grdina DJ, Basic I, Mason KA and Withers HR (1975) Radiation response of clonogenic cell populations separated from a fibrosarcoma. *Radiat Res* **63:** 483–493

Haimovitz-Friedman A, Kan CC, Ehleiter D, Persaud RS, Mcloughlin M, Fuks Z and Kolesnick RN (1994) Ionizing radiation acts on cellular membranes to generate ceramide and initiate apoptosis. *J Exp Med* **180:** 525–535

Hakomori S (1981) Glycosphingolipids in cellular interaction, differentiation and oncogenesis. *Annu Rev Biochem* **50:** 733–764

Hall EJ (1972) Radiation dose rate: a factor of importance in radiobiology and radiotherapy. *Br J Radiol* **45:** 81–97

Hill HZ, Hill GJ, Miller CF, Kwong F and Purdy J (1979) Radiation and melanoma: response of B16 tumor cells and clonal lines *in vitro*. *Radiat Res* **80:** 259–276.

Hosoi Y and Sakamoto K (1993) Suppressive effect of low dose total body irradiation on lung metastasis: dose dependency and effective period. *Radiother Oncol* **26:** 177–179

Jacubovich R and Doré JF (1979) Tumor associated antigens in culture medium of malignant melanoma cell strains. *Cancer Immunol Immunother* **7:** 59–64

Jenkins VK, Barranco SC, Townsend CM, Perry RR and Ives KL (1986) Differential response to gamma radiation of human stomach cancer cells *in vitro*. *Int J Radiat Biol* **50:** 269–278

Kono K, Tsuchida, T Kern, DH and Irie R (1990) Ganglioside composition of human melanoma and response to antitumor treatment. *Cancer Invest* **8:** 161–167

Lambin P, Marples B, Fertil B, Malaise EP and Joiner MC (1993) Hypersensitivity of a human tumor cell line to very low radiation doses. *Int J Rad Biol* **63:** 639–650

Leith JT, Dexter DL, Dewyngaert JK, Zeman EM, Chu MY, Calabresi P and Glicksman AS (1982) Differential responses to X-irradiation of subpopulations of two heterogeneous human carcinomas *in vitro*. *Cancer Res* **42:** 2556–2561

Li R, Villacreses N and Ladisch S (1995) Human tumor gangliosides inhibit murine immune responses *in vivo*. *Cancer Res* **55:** 211–214

Liotta LA (1990) Cancer invasion and metastases. *J Am Med Assoc* **263:** 1123–1126

Loeffler JS, Harris JR, Dahlberg WK and Little JB (1990) *In vitro* radiosensitivity of human diploid fibroblasts derived from women with unusually sensitive clinical responses to definitive radiation therapy for breast cancer. *Radiat Res* **121:** 227–231.

Mothersill C, Harney J, Lyng F, Cottel D, Parsons K, Murphy DM and Seymour CB (1995) Primary explants of human uroepithelium show an unusual response to low-dose irradiation with cobalt-60 gamma rays. *Radiat Res* **142:** 181–187.

Neri A and Nicolson GL (1981) Phenotypic drift of metastatic and cell-surface properties of mammary adenocarcinoma cell clones during growth *in vitro*. *Int J Cancer* **28:** 731–738

Portoukalian J and Bouchon B (1986) Hydrolysis of all gangliosides, including GM1 and GM2, on thin-layer plates by *Vibrio cholerae* neuraminidase. *J Chromatogr* **380:** 386–392

Portoukalian J, Carrel S, Doré, JF and Römke P (1991) Humoral immune response in disease-free advanced melanoma patients after vaccination with melanoma-associated gangliosides. *Int J Cancer* **49:** 893–899

Puck TT and Marcus PI (1956) Action of X-rays on mammalian cells. *J Exp Med* **103:** 63–669

Ramakrishnan N, Mcclain DE and Catravas GN (1993) Membranes as sensitive targets in thymocyte apoptosis. *Int J Rad Biol* **63:** 693–701

Rantanen V, Grénman S, Kulmala J, Jaakkola M, Lakkala T Sajantila A, Klemi P and Grénman R (1995) Characterization and radiosensitivity of UT-EC-2A and UT-EC-2B, two new highly radiosensitive endometrial cancer cell lines derived from a primary and metastatic tumor of the same patient. *Gynecol Oncol* **56:** 53–62

Rofstad EK (1992) Radiation sensitivity *in vitro* of primary tumors and metastatic lesions of malignant melanoma. *Cancer Res* **52:** 4453–4457

Suit H, Allam A, Allalunis-Turner J, Brock W, Girinsky T, Hill S, Hunter N, Milas L, Pearcey R, Peters L, Welch DR, West C and Efird J (1994) Is tumor cell radiation resistance correlated with metastatic ability? *Cancer Res* **54:** 1736–1741

Thomas CP, Buronfosse A, Portoukalian J and Fertil B (1995*a*) Correlation between the radiosensitivity *in vitro* of clones and variants derived from a human melanoma cell line and their spontaneous metastatic potential *in vivo*. *Cancer Lett* **88:** 221–225

Thomas CP, Buronfosse A, Fertil B and Portoukalian J (1995*b*) Surface expression of GD3 disialogangliosides in human melanoma cells is correlated to both metastatic potential *in vivo* and radiosensitivity *in vitro* CR *Acad Sci Paris* **318:** 1233–1238

Thomas CP, Buronfosse A, Combaret V, Pedron S, Fertil B and Portoukalian J (1996) Gangliosides protect human melanoma cells from ionizing radiation-induced clonogenic cell death. *Glycoconjugate J* **13:** 377–384.

Uzawa A, Susuki G, Nakata Y, Akashi M, Ohyama H and Akanuma A (1994) Radiosensitivity of CD45RO$^+$ memory and CD45RO$^-$ naive cells in culture. *Radiat Res* **137:** 25–33.

Welch DR, Milas L, Tomasovic SP and Nicolson GL (1983) Heterogeneous response and clonal drift of sensitivities of metastatic 13762NF mammary adenocarcinoma clones to γ-radiation *in vitro*. *Cancer Res* **43:** 6–10

 © Cancer Research Campaign 1997

Yang X, Darling JL, MC Millan TJ, Peacock JH and Steel GG (1991) Heterogeneity of radiosensitivity in a glioma cell line. *Int J Rad Oncol Biol Phys* **22:** 103–108

Zebda N, Bailly M, Brown S, Doré JF and Berthier-Vergnes O (1994) Expression of PNA-binding sites on specific glycoproteins by human melanoma cells is associated with a high metastatic potential. *J Cell Biochem* **54:** 161–173

Zebda N, Pedron S, Rebbaa A, Portoukalian J and Berthier-Vergnes O (1995) Deficiency of ganglioside biosynthesis in metastatic human melanoma cells: relevance of CMP-NeuAc: Lac Cer α2–3 sialyltransferase (GM3 synthase) *FEBS Lett* **362:** 161–164

© Cancer Research Campaign 1997